# Aggregation‐Induced Emission Photosensitizer Boosting Algal Growth and Lipid Accumulation

**DOI:** 10.1002/smll.202402463

**Published:** 2024-08-19

**Authors:** Sharmin Rakhi, AHM Mohsinul Reza, Brynley Davies, Jianzhong Wang, Youhong Tang, Jianguang Qin

**Affiliations:** ^1^ College of Science and Engineering Flinders University Bedford Park 5042 Australia; ^2^ Institute for NanoScale Science and Technology Flinders University Bedford Park 5042 Australia; ^3^ College of Veterinary Medicine Jilin Agricultural University Changchun 130118 China

**Keywords:** aggregation‐induced emission, light wavelength, lipid, microalgae, photosensitiser, reactive oxygen species

## Abstract

Mass production of microalgae is a research focus owing to their promising aspects for sustainable food, biofunctional compounds, nutraceuticals, and biofuel feedstock. This study uses a novel approach to enhance microalgae‐derived biomass and metabolites by using an aggregation‐induced emission (AIE) photosensitizer (PS), CN‐TPAQ‐PF_6_ ([C_32_H_23_N_4_]^+^). The unique AIE features of CN‐TPAQ‐PF_6_ facilitate nano‐aggregation in aquatic media for an effective light spectral shift for photosynthetic augmentation in a green microalga, *Chlamydomonas reinhardtii*. The high reactive oxygen species (ROS) production capacity and redox‐based cellular modulations reveal its potential to upsurge algal growth and lipid biosynthesis and fabricate fatty acid profiles in the metabolic pathways. Algal cells are labeled with other AIE‐based nanoprobes, which are suitable as an in vivo visualization toolkit with superior fluorescence. Furthermore, cytotoxicity analysis of CN‐TPAQ‐PF_6_ on the HaCat cell line confirms that this AIE PS is biocompatible without adverse impact on living cells. The results demonstrate the property of AIE PS for the first time in enhancing algal growth and lipid accumulation simultaneously.

## Graphical abstract

Photosensitizer (PS) mediated growth and lipid production in *Chlamydomonas reinhardtii*. Aggregation‐induced emission (AIE) photosensitizer (PS), CN‐TPAQ‐PF_6_ converts green light to red light and generates ROS. Higher energetic photon stimulates e^‐^ flow to increase ATP and NADPH production. NADPH provides reducing power for lipid synthesis. ROS induces lipid production through the activation and regulation of different pathways.

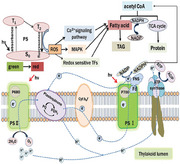

## 1 Introduction

Photosynthetic microalgae have attracted research recently for their essential biomolecules, specifically for the third and fourth‐generation feedstock for biofuel.^[^ ^1^, ^2^, ^3^ ^]^ Biofuel from single‐cell microalgae is considered a promising alternative to stock‐limited fossil fuels for the recent price hike and future demand and for minimizing carbon emissions with 10 to 50 times higher efficiency than terrestrial plants.^[^ ^4^, ^5^ ^]^ Furthermore, microalgae‐derived polyunsaturated fatty acids (PUFAs) have various possible health‐promoting functions with higher acceptability than PUFAs from other marine fish and shellfish due to product quality and safety.^[^ ^6^, ^7^ ^]^ Apart from lipids, microalgae are an excellent source of different metabolites for biomedical and pharmaceutical applications. However, industry‐scale microalgae culture for lipid and biomass production is still a challenge. The lipid content in most microalgae can be maximized by applying physico‐chemical stressors or nutrient starvation, which eventually inhibits cellular growth.^[^ ^8^, ^9^, ^10^ ^]^ Despite promising aspects, genetically engineered microalgal strains are associated with market acceptability and potential environmental hazards in an open culture system.^[^ ^11^ ^]^

Efficient light energy conversion during photosynthesis can promote the biomass yield and their high‐valued reaction products. The main photosynthetic pigments in green microalgal photosystem are chlorophyll *a* and *b*, which are excited at blue (chlorophyll *a*: 440 nm, chlorophyll *b*: 435 nm) and red region (chlorophyll *a*: 660 nm, chlorophyll *b*: 643 nm) of photosynthetically active radiation (PAR, 400–700 nm).^[^ ^12^, ^13^ ^]^ Spectral conversion to the acceptable range of light‐harvesting molecules is an effective mechanism for the augmentation of photosynthesis. Some conventional luminogens have been investigated for growth and lipid production as spectral shift materials. However, partial light conversion, auto‐absorption, strong background noise, low photostability, cytotoxicity in direct contact, and weak fluorescence in solution due to aggregation‐caused quenching (ACQ) eventually have limited their application.^[^ ^14^, ^15^ ^]^ Therefore, an effective fluorescent molecule that minimizes these drawbacks is needed to scale up industry‐scale microalgal production.

With the discovery of the aggregation‐induced emission (AIE) phenomenon, several AIE photosensitizers (PS) have been synthesized with unique fluorescence properties and efficient energy transfer capacities.^[^ ^16^, ^17^ ^]^ AIE PS are excited in the ground singlet state (S_0_) to a nearby higher energy state and then relax in the lowest energy state (S_1_) by releasing energy or superior luminescence via non‐radiative decay and restricting the intramolecular motion (RIM) in the aggregated excited state. The longer and energetic T_1_ allows PS to interact with surrounding molecules to produce type I like superoxide radicals (O_2_^•−^), hydrogen peroxide (H_2_O_2_), and hydroxyl radicals (HO^•−^) by an electron transfer mechanism, and type II ROS as singlet oxygen (^1^O_2_) by an energy exchange mechanism. Different synthesis processes can further trigger light harvesting capacity and type I and type II ROS generation.^[^ ^18^, ^19^ ^]^ ROS are considered detrimental as they may cause cellular alteration at higher concentrations, leading to oxidative damage, organelle dysfunction, and mutagenesis.^[^ ^20^ ^]^ However, recent studies suggest that certain ROS levels can act as signaling molecules in fundamental biological processes in different organisms.^[^ ^21^, ^22^ ^]^

In this study, we used an AIE PS, CN‐TPAQ‐PF_6_ ([C_32_H_23_N_4_]^+^) to investigate its effect on the enhancement of biomass and metabolic reaction products of a green microalga, *Chlamydomonas reinhardtii*. Light harvesting capacity and high ROS generation of CN‐TPAQ‐PF_6_ were previously reported.^[^ ^18^ ^]^ The generated ROS by photosensitizers can be subsequently alleviated by the action of different enzymatic (e.g., ascorbate peroxidase, superoxide dismutase, and catalase) and non‐enzymatic (e.g., carotenoids, ascorbate, tocopherols, glutathione) antioxidants.^[^ ^23^, ^24^ ^]^ This study reveals that the AIE PS, CN‐TPAQ‐PF_6_ ([C_32_H_23_N_4_]^+^), can simultaneously enhance algal growth and lipid accumulation without harming living cells.

## 2 Results and Discussion

### 2.1 Photophysical Properties of CN‐TPAQ‐PF_6_

**Figure 1:**
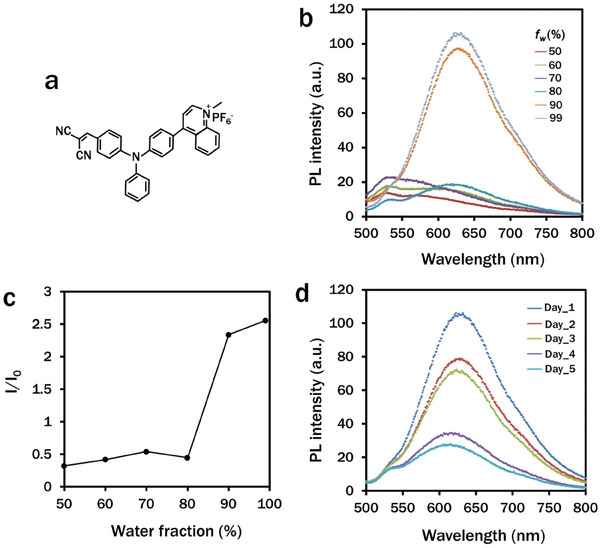
Photophysical properties of CN‐TPAQ‐PF_6_. a) The molecular structure of CN‐TPAQ‐PF_6_ adapted from Yu et al.^[^ ^16^ ^]^ b) and c) fluorescence spectra of CN‐TPAQ‐PF_6_ at different water fractions (*f_w_*), and d) fluorescence spectra of CN‐TPAQ‐PF_6_ on different days.

CN‐TPAQ‐PF_6_ was synthesized according to previously reported work (supporting information).^[^ ^18^ ^]^ The normalized photoluminescence (PL) intensity of 10 µM CN‐TPAQ‐PF_6_ in DMSO‐water mixture at 0–99% water fraction (*f_w_*) was recorded from 550 to 750 nm upon excitation at 500 nm with an emission peak at 650 nm (**Figure** **1**b). The emission intensities of CN‐TPAQ‐PF_6_ were totally quenched up to 70% *f_w_* before a dramatic amplification at 99% *f_w_* (Figure 1b). The rapid increase of the PL intensity with *f_w_* increase from 80% to 99% (Figure 1b) was due to the nano‐aggregation of CN‐TPAQ‐PF_6_ in DMSO‐water mixture without ACQ exhibiting its typical AIE properties. The large bathochromatic shift from 520 to 630 nm with increased solvent polarity demonstrated the strongly twisted intramolecular charge transfer (TICT) effect. The spectrophotometric analysis of the PL intensity of 10 µM CN‐TPAQ‐PF_6_ in DMSO‐water mixtures (99% *f_w_*) on different consecutive days suggested that the CN‐TPAQ‐PF_6_ remained functionality for five days (Figure 1d).

Due to the inverse relation of energy and wavelength, green light exhibits higher energy than red light. Thus, the light wavelength shift from green to red provides additional energy for photosynthetic augmentation by increasing chlorophyll *a* and *b* content in the red region.^[^ ^25^ ^]^ Upon excitation at 500 nm, the photon of green light converted to orange‐red light with an emission at 600–700 nm for absorption by chlorophyll *a* and *b* in the reaction centers, providing sufficient photon energy for intra‐molecular charge separation within the electron transport chain of the thylakoid lumen. Different conventional luminogens have been reported as light spectral shift materials with low biomass yield at indirect contact with algae and plant cells.^[^ ^26^, ^27^ ^]^ Most of these luminogens have overlapped excitation and emission wavelengths with minimum stroke shift, leading to the self‐absorption of fluorescence that fails to play a substantial role in the effective spectral shift. CN‐TPAQ‐PF_6_ exhibited clearly distinguishable absorption (488–580 nm) and emission spectra (600–700 nm) with a larger stroke shift of 110 nm, eventually revealing its potential as a highly efficient AIE‐active light shifting PS.

### 2.2 H_2_O_2_ Generation of CN‐TPAQ‐PF_6_ in *C. reinhardtii* Cell

Promising ROS generation was previously demonstrated, especially type I ROS production with CN‐TPAQ‐PF6.^[^ ^16^ ^]^ Herein, as a ROS molecule, H_2_O_2_ generation at different concentrations of CN‐TPAQ‐PF_6_ was investigated in algal cells with confocal microscopy. Compared to the other redox‐based molecules, the stability of H_2_O_2_ advantages its movement through different membranes for effective modulation in signaling pathways. Furthermore, the involvement of H_2_O_2_ in the regulation of the various systems, including the activation of growth and development regulatory genes (eg. *bZIP* gene), and other modulations of secondary metabolism, are relatively well explored in microalgae.^[^ ^28^, ^29^ ^]^

**Figure 2:**
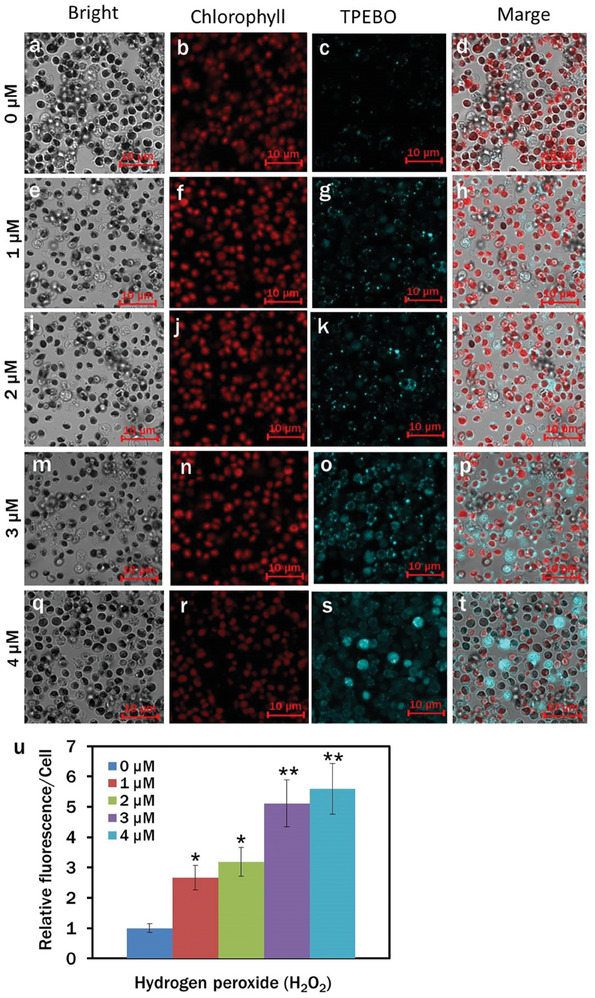
a–t) Confocal images of H_2_O_2_ generation in *C. reinhardtii* cells at different concentrations of CN‐TPAQ‐PF_6_. Cells were stained with 100 µM TPE‐BO followed by 20 min incubation in the dark. Fluorescence images‐Bright: a), e), i), m), and q); chlorophyll: b), f), j), n), and r), (*λ*_ex_: 488 nm, *λ*_em_: 685–758 nm); TPE‐BO: c), g), k), o), and s), *(λ*_ex_: 405 nm, *λ*_em_: 428–499 nm); merged: d), h), l), p), and t); u) relative fluorescence/cell for different H_2_O_2_ accumulation. Averages are shown as mean ± SE; (*p < 0.05, **p < 0.01).

An AIE‐based H_2_O_2_ specific nanoprobe, TPE‐BO (C_38_H_42_B_2_O_4_, *λ*_ex_: 405 nm, *λ*_em_: 428–499 nm) was synthesized and employed to detect the H_2_O_2_ inside the algal cells. The PL properties and the H_2_O_2_‐specificity of TPE‐BO were previously characterized.^[^ ^30^ ^]^ In the control, H_2_O_2_ accumulation was insignificant in *C. reinhardtii* cells, as the tranquil blue TPE‐BO channel had the least fluorescence upon excitation at the appropriate wavelength (**Figure** **2**c). The *C. reinhardtii* cells cultured with 1, 2, and 3 µM CN‐TPAQ‐PF_6_ exhibited higher H_2_O_2_ accumulation than the control with superior fluorescence Figure 2g,k,o). The maximum H_2_O_2_ accumulation was observed in 4 µM CN‐TPAQ‐PF_6_ treated cells in the respective channel (Figure 2s). Relative fluorescence/cell at 0, 1, 2, 3, and 4 µM CN‐TPAQ‐PF_6_ revealed that H_2_O_2_ accumulation within algal cells was associated with PS concentration.

### 2.3 Effects of CN‐TPAQ‐PF_6_ on Growth and Biomass of *C. reinhardtii*

**Figure 3:**
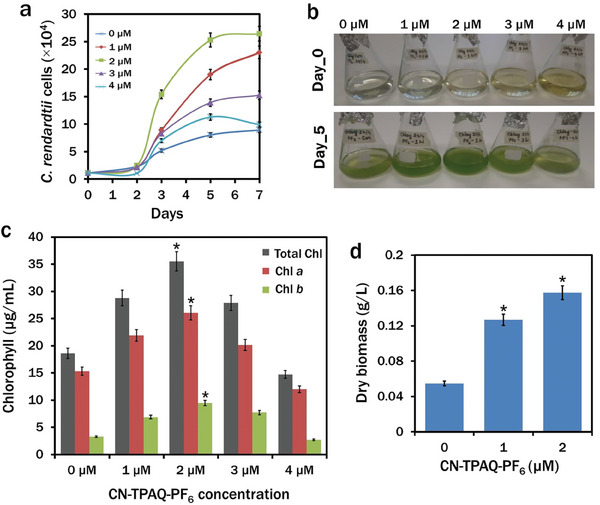
Effects of CN‐TPAQ‐PF_6_ on growth, biomass, and chlorophyll of *C. reinhardtii*. a) growth curve of *C. reinhardtii* at different concentrations of CN‐TPAQ‐PF_6_ for 7 days, b) *C. reinhardtii* culture at day‐0 and day‐5; c) total chlorophyll, chlorophyll *a* and *b* (µg mL^−1^) production at different concentrations of CN‐TPAQ‐PF_6_ in *C. reinhardtii* at day‐5; d) biomass (g L^−1^) production at 0, 1, and 2 µM CN‐TPAQ‐PF_6_ exposed cells. Data are presented as mean ± SE (*p < 0.05).

To determine the effect of CN‐TPAQ‐PF_6_ on the growth and biomass of *C. reinhardtii*, cells were cultured with 0 (control), 1, 2, 3, and 4 µM CN‐TPAQ‐PF_6_ for 7 days. Initial growth remained steady on day 1 and day 2 with a slight decline at 4 µM CN‐TPAQ‐PF_6_ exposed to culture, possibly due to the ROS‐mediated stress (**Figure** **3**a). The rapid acceleration of growth in each treatment was recorded for the following days with a maximum growth of 25 ± 1.38 (10^4^) cells mL^−1^ in 2 µM CN‐TPAQ‐PF_6_ treated cells followed by 3 and 4 µM CN‐TPAQ‐PF_6_ treated cells (Figure 3a,b). The photosynthetic apparatuses, chlorophyll *a* and *b* in all cultures were recorded at the growth phase of 5 days. A significant increase was observed in both chlorophyll *a* (26.06 ± 0.91 µg mL^−1^) and *b* (9.47 ± 0.21 µg mL^−1^) content in 2 µM CN‐TPAQ‐PF_6_ treated cells (Figure 3c). Biomass from 0 µM (control), 1 µM and 2 µM CN‐TPAQ‐PF_6_ exposed cultures showed significant (^*^ *p* < 0.5) increase in both 1 and 2 µM CN‐TPAQ‐PF_6_ exposed biomass with approximately three‐fold higher biomass production in 2 µM than that of the control (Figure 3d).

In the synthesis process of CN‐TPAQ‐PF_6_, conjugation of triphenylamine (TPA) with quinolinium hexafluorophosphate (PF_6_) group provided strong acceptor–donor–acceptor (A–D–Aʹ) type to facilitated intramolecular charge transfer (ICT) for more extended emission range (orange to red) whereas, cyano addition and cationization improved light harvesting and cellular binding capacities respectively,^[^ ^18^ ^]^ providing a potential approach for photosynthetic pigments to absorb more energy in the red region for algal growth and lipid biosynthesis. This higher entrapped energy enters the electron transport chain in the thylakoid lumen to produce the subsequent amount of adenosine triphosphate (ATP) and reduce power in terms of nicotinamide adenine dinucleotide phosphate hydrogen (NADPH) for biomolecule synthesis in the presence of light. We maintained *C. reinhardtii* culture in a continuous light condition that was likely to enact Ferredoxin: NADP^+^ reductase (FNR) for the effective electron cycling from ferredoxin to NADP since the activity of FNR is inhibited in the dark.^[^ ^31^ ^]^

Microalgal growth enhancement with other AIE‐based light‐shifting nanoparticles has been reported.^[^ ^32^, ^33^ ^]^ However, the effect of AIE PS and ROS‐mediated microalgal growth has not been previously reported. ROS are inevitable molecules for plant growth and development. In vitro, the addition of H_2_O_2_ was reported to improve the wall formation in cotton, and H_2_O_2_ and O_2_^−^ were associated with beta‐ureidopropionase 1 (*UPB1*) expression, which in turn can regulate growth and cell wall remodeling.^[^ ^22^ ^]^ Although in *C. reinhardtii*, different ROS molecules are associated with the activation and regulation of Ca^2+^ signaling pathways, the exact mechanism of Ca^2+^ signaling toolkit and its homeostasis is still limited and needs to be elucidated.^[^ ^34^ ^]^ Ca^2+^ signal transduction and redox regulation are responsible for the activation and modulation of photosynthetic cyclic electron flow (CEF), which maintains the pH‐gradient across the thylakoid membrane via the NAD(P)H dehydrogenase (NDH)‐dependent pathway for effective photosynthesis.^[^ ^35^ ^]^ The Ca^2+^ network in *C. reinhardtii*, Ca^2+^ sensor protein (CAS) controls the expression of Light‐Harvesting Complex Stress Related 3 (LHCSR3) during photoacclimation to maintain a basal metabolism.^[^ ^36^ ^]^ Due to the involvement of different pathways and signal transduction systems, H_2_O_2_ can enhance growth in microalgae.

### 2.4 Performance of the Lipid Specificity of 2‐DPAN

**Figure 4:**
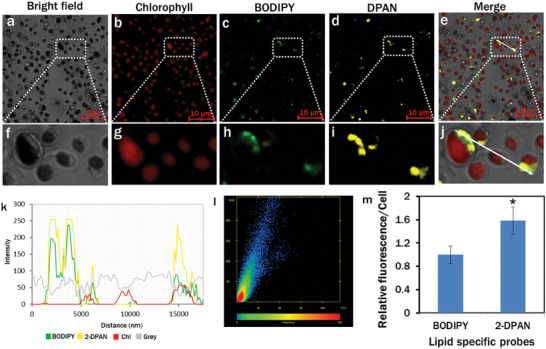
Performance of the lipid specificity of AIE‐based lipid‐specific probe, 2‐DPAN. a–j) Confocal images of lipid drops in *C. reinhardtii* cells. Cells were labeled with traditional BODIPY™ 505/515 (10 µM; 20 min incubation) and 2‐DPAN (20 µM; 20 min incubation). Fluorescence images a) bright field; (b) chlorophyll: *λ*_ex_: 488 nm, *λ*_em_: 685–758 nm; c) BODIPY: *λ*_ex_: 488 nm, *λ*_em_: 490–517 nm; d) 2‐DPAN: *λ*_ex_: 488 nm, *λ*_em_: 570–650 nm; e) merge; k) intensity profile of BODIPY and 2‐DPAN in green and yellow channels; l) intensity scatter plot for the colocalized channels; Pearson correlation coefficient and Mander's overlap coefficient were calculated as 0.90 and 0.91, respectively; m) Relative fluorescence intensity/cell. Values are relative to the control BODIPY dye. Data are presented as mean ± SE; (*p < 0.05).

Confocal images of *C. reinhardtii* cells were used to determine the performance of two lipid‐specific probes, the conventional BODIPY 505/515 and AIE‐based 2‐DPAN (C_24_H_18_N_2_O). BODIPY was obtained commercially, and 2‐DPAN was synthesized using the method previously described (supporting information).^[^ ^34^ ^]^ Fluorescence of BODIPY *(λ*_ex_: 488 nm, *λ*_em_: 490–517 nm) and 2‐DPAN (*λ*_ex_: 488 nm, *λ*_em_: 570–650 nm) was observed in green (**Figure** **4**c) and yellow channels (Figure 4d), respectively. The arrow in the merged image (Figure 4e,j) exhibits the synchronized intensity changes of BODIPY and 2‐DPAN. The intensity profile of BODIPY in the green channel was colocalized with the yellow channel of 2‐DPAN (Figure 4k). The Pearson's correlation coefficient and Mander's overlap coefficient were calculated as 0.90 and 0.91 from the intensity scattered plot (Figure 4l). Relative fluorescence intensity/cell demonstrated 1.5‐fold superior fluorescence of 2‐DPAN than BODIPY (Figure 4m). Being an AIE‐based nano‐probe, 2‐DPAN is free of ACQ in aquatic media. Additionally, the keto‐salicylaldehyde hydrazine (KSA) in 2‐DPAN increased hydrophobicity and restricted intramolecular motion to enhance brightness.^[^ ^37^ ^]^ Therefore, in the present study, 2‐DPAN was further used for in vivo lipid labeling.

### 2.5 CN‐TPAQ‐PF_6_ Induced Lipid Accumulation and Fatty Acids Proliferation

**Figure 5:**
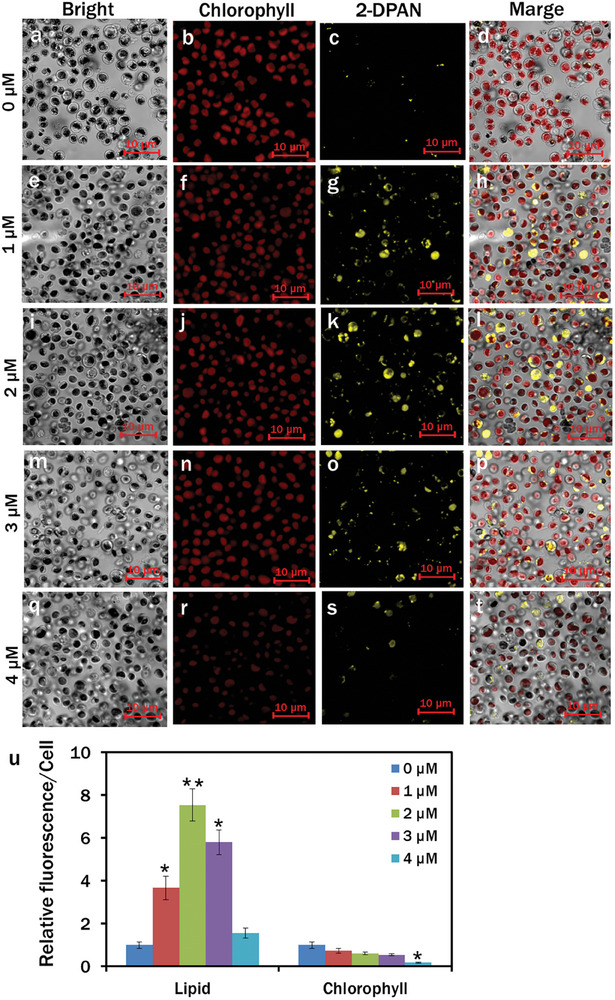
Confocal images of lipid drops in *C. reinhardtii* cells. Cells were labeled with 2‐DPAN (20 µM; 20 min incubation) fluorescence images: bright field – a), e), i), m), q); chlorophyll: (*λ*_ex_: 488 nm, *λ*_em_: 685–758 nm) – b), f), j), n), r); 2‐DPAN (*λ*_ex_: 488 nm, *λ*_em_: 570–650 nm) – c), g), k), o), s); merge‐ d), h), l), p), t). u) Relative fluorescence intensity/cell. Values are relative to the control. Data are presented as mean ± SE; (*p < 0.05, **p < 0.01).

**Figure 6:**
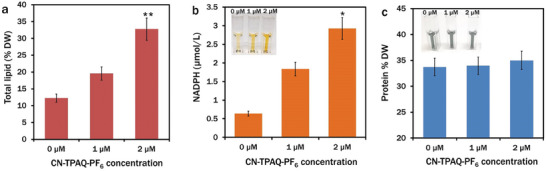
Effect of CN‐TPAQ‐PF_6_ on different cellular components of *C. reinhardtii*. a) Total lipid production (% DW) at different CN‐TPAQ‐PF_6_ concentrations. b) Total NADPH µmol L^−1^ at different CN‐TPAQ‐PF_6_ concentrations; Inset: colorimetric change due to the presence of different NADPH concentrations. c) Total protein content (% DW) at different concentrations of CN‐TPAQ‐PF_6_; inset: insignificant colorimetric change due to the presence of the same protein concentration in different treatments. Data are presented as mean ± SE; (*p < 0.05, **p < 0.01).

Herein, CN‐TPAQPF_6_ induced lipid accumulation in cells cultured for 7 days was determined by confocal analysis labeling the cells with 2‐DPAN. Total lipid and fatty acid methyl esters (FAME) were measured from freeze‐dried samples of 7‐day cultured cells. Confocal analysis revealed the distinguished lipid accumulation pattern in different treatment groups of *C. reinhardtii* cells. Maximum lipid production occurred in 2 µM CN‐TPAQ‐PF_6_ exposed cells with the highest fluorescence in the 2‐DPAN channel (**Figure** **5**k). Lipid accumulation started to decline at 3 µM CN‐TPAQ‐PF_6_ exposed cells (Figure 5o), and the least amount of lipid was observed in 4 µM CN‐TPAQ‐PF_6_ treated cells with reduced fluorescence in the respective channel (Figure 5s). Relative fluorescence/cell showed a significant increase (^*^ *p* < 0.05, ^**^ *p* < 0.01) in lipid accumulation in 1, 2, and 3 µM CN‐TPAQ‐PF_6_ treated cells (Figure 5u). This result revealed that CN‐TPAQ‐PF_6_ concentrations and associated H_2_O_2_ generation triggered the lipid biosynthesis and maximized the production at a certain concentration. A significant decrease (^*^ *p* < 0.05) in chlorophyll content in 4 µM CN‐TPAQ‐PF_6_ treated cells (Figure 5u) was possibly due to excessive H_2_O_2_ generation at that concentration. The chlorophyll content in different treatment groups on day 7 was lower than on day 5 (Figure 3c), suggesting its utilization as reserved nitrogen for stress alleviation during lipid biogenesis.^[^ ^38^, ^39^ ^]^ Moreover, adequate ATP reserve due to the PS‐mediated efficient light utilization might facilitate further rapid chlorophyll synthesis from glutamate precursor to maintain homeostasis at a certain level. In comparison to the control, an almost three‐fold upturn in total crude lipid production was observed in 2 µM CN‐TPAQ‐PF_6_ exposed 7‐day culture cells and measured as 12.24 ± 2.55% dry weight and 32.77 ± 5.92% dry weight, respectively (**Figure** **6**a).

Among the fatty acids (FAs) groups, saturated fatty acids (SFAs) upsurged by ≈16.09% in comparison to that of control and dominated by palmitic acid (C16:0) and margaric acid (C17:1), the precursors of biodiesel production (**Table** **1**). Increased accumulation of health beneficiary oleic acid (C18:1) and α‐linoleic acid (C18:3) was outweighed in mono and polyunsaturated groups (MUFAs and PUFAs) (Table 1).

**Table 1:** Percentage of FAMEs in *Chlamydomonas reinhardtii* cells under different cultural conditions.

FAMEs [%]	CN–TPAQ–PF_6_ Treatments		
	0 µM	1 µM	2 µM
C8:0	0.32 ± 0.18	2.07 ± 0.15	2.92 ± 0.28
C10:0	0.12± 0.14	0.13 ± 0.21	0.18 ± 0.12
C11:0	0.11 ± 0.04	0.16 ± 0.11	0.16 ± 0.03
C12:0	–*	0.12 ± 0.07	0.15 ± 0.17
C13:0	0.10 ± 0.02	0.12 ± 0.03	0.13 ± 0.02
C14:0	0.19 ± 0.05	0.30 ± 0.02	0.42 ± 0.06
C14:1	0.15 ± 0.02	0.38 ± 0.09	0.88 ± 0.21
C15:0	–*	–*	0.12 ± 0.01
C15:1	0.15 ± 0.08	1.05 ± 0.14	3.29 ± 0.32
C16:0	16.30 ± 1.25	19.89 ± 1.97	28.09 ± 2.83
C16:1	7.01 ± 0.52	4.80 ± 0.74	2.63 ± 1.19
C17:0	6.29 ± 1.32	4.81 ± 0.62	7.12 ± 1.62
C17:1	10.53 ± 0.87	11.15 ± 1.73	16.17 ± 2.02
C18:0	4.02 ± 0.62	2.13 ± 0.68	3.4 ± 0.32
C18:1	12.15 ± 0.92	13.94 ± 2.12	13.3 ± 2.77
C18:2	16.65 ± 1.93	14.34 ± 1.72	10.56 ± 2.08
C18:3	16.02 ± 1.68	19.12 ± 2.14	23.65 ± 2.71
C20:0	0.31 ± 0.02	0.20 ± 0.01	0.17 ± 0.01
C20:1	0.72 ± 0.05	0.58 ± 0.07	–*
C20:3	0.38 ± 0.04	0.32 ± 0.03	0.32 ± 0.01
C23:0	–*	–*	0.48 ± 0.01
C24:0	–*	0.29 ± 0.01	1.86 ± 0.06
C24:1	–*	–*	2.72± 0.74
SAFAs	39.76 ± 6.29	40.05 ± 7.05	46.75 ± 7.95
MUFAs	23.69 ± 4.01	22.8 ± 5.05	24.28 ± 3.05
PUFAs	32.67 ± 5.01	31.46 ± 3.05	30.45 ± 4.03

* Non‐detectable.

Direct employment of H_2_O_2_ in the culture has also been reported to enhance the yield of pigments like astaxanthin, lipid, and FAs accumulation in microalgae with associated growth decline.^[^ ^40^, ^41^ ^]^ Although this study exhibited a promising upsurge in lipid and FAs production, the exact mechanisms could not be fully elucidated due to the complex regulatory pathways of lipid biogenesis in microalgal cells.^[^ ^42^ ^]^ ROS generation, regulatory mechanisms, and involvement in different biological processes have not been completely understood in microalgae.^[^ ^34^ ^]^ However, H_2_O_2_ may activate calcium ion (Ca^2+^) signals through the direct activation of the receptor‐like protein kinases (RLKs), which triggers the activation of mitogen‐activated protein kinase (MAPK) cascade by generating phosphatidic acid (PA).^[^ ^43^ ^]^ Thus, the crosstalk of H_2_O_2_ and Ca^2+^ stimulate MAPK mediated phosphorylation to regulate transcription factors (TFs) for further respective gene expression such as *BCCP2* gene encoding an *ACCase* and *At3955310* gene encoding ketoacyl‐ACP reductase for FAs biosynthesis.^[^ ^44^ ^]^

### 2.6 Cellular NADPH and Its Role in Lipid Biosynthesis

In microalgae, harvested photon produces energy in the form of ATP and reduce power in NADPH in photosystems I and II of the thylakoid lumen, which is further utilized in different cellular metabolisms. Herein, we determined the cellular NADPH in a 5‐day culture of *C. reinhardtii* with different concentrations of CN‐TPAQ‐PF_6_. Compared to the control, a significant increase in total NADPH was recorded from 2 µM CN‐TPAQ‐PF_6_ exposed culture and measured as 0.6 ± 0.006 and 2.9 ± 0.5 µmol L^−1^, respectively (Figure 6b).

The light‐harvesting capacity and effective light conversion of CN‐TPAQ‐PF_6_ facilitate much energy entrapment in photosynthetic machinery (PSII and PSI) for high energy electron transport in ferredoxin of PSI to produce NADPH by Ferredoxin NADP^+^ reductase (FNR) in the presence of light.^[^ ^45^ ^]^ NADPH and ATP enhanced acetyl‐CoA supply in the lipid synthesis cascade, then polymerized during *de novo* lipogenesis for FAs and triacylglycerols (TAGs) synthesis.^[^ ^46^ ^]^ Also, the cellular NADPH is catalyzed by cytoplasmic NADPH oxidase to transfer electrons for dismutation of O_2_^−^ to H_2_O_2_ for further lipid biosynthesis.

Interestingly, protein content followed a similar pattern in different treatment groups (Figure 6c). This is possibly due to the continuous supply of acetyl‐CoA for minimal competition between protein and FA synthesis pathways, which utilize the acetyl‐CoA as a common precursor molecule. Additionally, in the ROS gene network of *C. reinhardtii*, the respiratory burst oxidase homolog (RBOH) encoded gene maintains the basal ROS level for redox biology by producing ascorbate peroxidase (APX), catalase (CAT), glutathione peroxidase (GPX) and peroxiredoxin (PRXR).^[^ ^47^ ^]^ The appearance of these enzymes might be involved in the protein content of different treatment groups.

We also determined the cytotoxicity of 1 and 2 µM CN‐TPAQ‐PF_6_ on HaCaT cell lines and found strong biocompatibility of this PS with 100% cell viability (Figure S2, Supporting Information). Gas chromatography‐mass spectrometry (GCMS) analysis of 2 µM CN‐TPAQ‐PF_6_ on day 0 and day 7 showed that functional molecules of this PS were completely diminished after 7 days (Figures S3 and S4 Supporting Information). Therefore, the biocompatibility of this probe indicates its suitability for utilization in algal biofactories for human food and nutraceutical research.

## 3 Conclusion

This study discovered the synergic effects of an AIE photosensitizer, CN‐TPAQ‐PF_6_, on the growth and lipid proliferation of *C. reinhardtii* under laboratory conditions. We used the AIE‐based visualizing nanoprobes TPE‐BO and 2‐DPAN with superior fluorescence for effective labeling H_2_O_2_ and lipids respectively. Efficient light conversion to the acceptable range of chlorophyll and sufficient photostability of CN‐TPAQ‐PF_6_ triggered photosynthesis and optimum H_2_O_2_ generation at 2 µm concentration, inducing growth and photosynthetic synthesis of metabolites. The high biocompatibility of this nanoparticle provides its suitability for safe and sustainable microalgae‐based biomolecule production. As the effects of AIE PS on microalgal biomass and lipid enhancement are demonstrated for the first time, further investigations are needed to determine their commercial application.

## 4 Experimental Section

### Microalga Culture

A pure strain of *C. reinhardtii* was obtained from the Biology Discovery Centre, Flinders University, Australia. Culture was established in the modified Wood Hole (MBL) medium^[^ ^48^ ^]^ after autoclaving at 121 °C for 15 min. The 1‐L culture medium contained CaCl_2_.2H_2_O (0.04 g), MgSO_4_.7H_2_O (0.04 g), NaHCO_3_ (0.01 g), K_2_HPO_4_ (0.008 g), NaNO_3_ (0.08 g), EDTA‐Na (0.004 g), FeCl_3_.6H_2_O (0.004 g), CuSO_4_.5H_2_O (0.01 mg), ZnSO_4_.7H_2_O (0.02 g), CoCl_2_.6H_2_O (0.01 mg), MnCl_2_.4H_2_O (0.02 mg), Na_2_MoO_4_.2H_2_O (0.006 mg), C_63_H_88_CoN_14_O_14_P (Vitamin B_12_) (0.0005 mg), C_12_H_17_ClN_4_OS·HCl (Vitamin B1) (0.1 mg), C_10_H_16_N_2_O_3_S (Biotin) (0.0005 mg) and C_4_H_11_NO_3_, (Tris(hydroxymethyl)aminomethane) (0.25 g). The pH of the culture medium was adjusted to 7.2. The culture was maintained under continuous white light illumination (LED) with a light intensity of 50 µmol photons m^–2^ s^–1^. The culture was maintained at 25 °C with continuous rotation at 100 rpm. All required reagents for MBL culture medium were obtained from Thermo Fisher Scientific, Australia.

### Determination of Fluorescence Spectra of CN‐TPAQ‐PF_6_

The PL spectra of 10 µM CN‐TPAQ‐PF_6_ in DMSO‐water mixtures at 60–99% *f_w_* were determined using a fluorescence spectrophotometer (Cary Eclipse, MY17180002, Agilent Technologies, CA, USA) with quartz cuvettes of 1 cm path length. Spectrophotometric analysis of the fluorescence intensity profile of CN‐TPAQ‐PF_6_ at 99% *f_w_* was recorded on different days to determine the functional stability.

### Fluorescent Staining of H_2_O_2_ and Confocal Analysis

This experiment used AIE‐based nanoprobe TPE‐BO to detect the H_2_O_2_ inside the algal cells. TPE‐BO was synthesized according to a previously published report.^[^ ^30^ ^]^

For fluorescence staining, 1 mM stock solution of TPE‐BO was prepared with DMSO. Cells were adjusted to 10^6^ mL^−1^ and incubated with 100 µM of TPE‐BO for 20 min at dark. The final DMSO concentration was adjusted to 0.1%. Confocal analysis was performed under a Zeiss LSM 880 Airyscan confocal microscope using ZEN 2.6 software (Carl Zeiss, Australia). For confocal analysis, the excitation and emission of TPE‐BO were set at 405 nm and 428–499 nm, respectively.

### Growth and Biomass Analysis of *C. reinhardtii*

To determine the effect of CN‐TPAQ‐PF_6_ concentrations on the *C. reinhardtii* growth, 1.0 ± 0.001 (×10^4^) cells were introduced to the culture medium at pre‐defined conditions. Different CN‐TPAQ‐PF_6_ concentrations (1, 2, 3, and 4 µm) dissolved in DMSO were introduced directly to the *C. reinhardtii* culture as treatments. Culture with no CN‐TPAQ‐PF_6_ was used as control. All cultures were conducted with three replications (n = 3). The growth rate of *C. reinhardtii* was determined by counting the cells with a hemocytometer (Improved Marienfeld Neubauer, Germany) under a light microscope at regular intervals for 7 days.

For biomass analysis, the 7‐day culture of 1 L *C. reinhardtii* cells was centrifuged at 4000 × *g* for 5 mins. Cell suspension was stored at 180 °C for 24 h before freeze drying for 48 h. The freeze‐dried samples were weighed and immediately stored at −20 °C for further analysis.

### Chlorophyll a and b Measurement

According to a previous report, samples were prepared for chlorophyll a and b quantification.^[^ ^49^ ^]^ In brief, 5 ml of algal samples were mixed in the reagent‐graded solvent extract of 80% (v/v) acetone and 20% (v/v) methanol. Algal cells in the culture medium were centrifuged at 15 000 × *g* for 10 min followed by re‐suspension and centrifugation. The supernatants were kept at 4 °C, protecting them from light. The optical density of the supernatants was measured at 646.6, 663.6, and 750 nm against the blank (80% acetone/20% (v/v) methanol) using a GENESYS 150 UV‐vis spectrophotometer (Thermo Fisher Scientific Inc., Australia). Chlorophyll *a* and *b* content were calculated according to the extinction coefficients.^[^ ^50^ ^]^

### Measurement of Lipid Specificity of BODIPY and 2‐DPAN

Confocal analysis (Zeiss LSM 880 Airyscan confocal microscope, Carl Zeiss, Australia) (Carl Zeiss, Australia) of 2 µM CN‐TPAQ‐PF_6_ treated *C. reinhardtii* cells was used to determine the performance of 2‐DPAN over the conventional lipid specific probe, BODIPY 505/515. Samples were properly stained with BODIPY 505/515 and 2‐DPAN.^[^ ^51^ ^]^ Briefly, 1.0 mM stock solution of BODIPY 505/515 and 2‐DPAN were prepared with DMSO. The stocks were then diluted to 10 µm with DI water, adjusting the final concentration of DMSO at 0.1%. *C. reinhardtii* cells were adjusted at 10^6^ cells mL^−1^, and were stained with 200 µL of 10 µM BODIPY and 10 µM 2‐DPAN and vortexed at 100 rpm for 30 s. The stained samples were incubated for 20 min and protected from light before analysis.

### Fluorescent Staining of Lipid and Confocal Analysis

Before using the AIE‐based nanoprobe, 2‐DPAN as a lipid imaging tool, the lipid specificity and fluorescence intensity of 2‐DPAN were determined and compared with BODIPY 505/515. The 7‐day cultured cells with different treatments were adjusted to 10^6^ and incubated with 20 µM 2‐DPAN for 30 min, avoiding light for confocal analysis. The excitation and the emission of 2‐DPAN were 488 and 526–570 nm, respectively. The autofluorescence of chlorophyll in *C. reinhardtii* was previously measured (*λ*_ex_:488 nm, *λ*_em_:685‐758 nm) (Figure S1, Supporting Information).

### Lipid Extraction and Fatty Acid Analysis

Microalgal lipids were extracted using an adapted method.^[^ ^52^, ^53^ ^]^ Lyophilised biomass from different treatments was weighed to 10 mg and dissolved in 50 µL of 1.5% NaCl buffer. An organic solvent (1 mL) of 2:1 chloroform and methanol (v/v) was then added and homogenized thoroughly for 10 min, followed by incubation at room temperature for 10 min. A further 200 µL of 1.5% NaCl buffer was added and homogenized thoroughly for 5 min. Phase separation was achieved by room temperature centrifugation at 6000 × *g* for 5 min. Of the organic phase, 500 µL was extracted and concentrated by nitrogen gas evaporation. Dried lipid extracts were determined gravimetrically and stored at −20 °C for further Gas Chromatography and Mass Spectrometry (GC‐MS) analysis of fatty acids.

For GC‐MS, dried lipid extracts were resuspended in 1:1 chloroform and trimethylsulphonium hydroxide (v/v) to generate fatty acid methyl esters (FAMEs). FAMEs were then subject to analysis using an Agilent 7890A GC system with a 30 m Agilent DB–FastFAME column (Agilent Technologies) coupled with an Agilent 5975C MSD system (Agilent Technologies) to complete mass spectrometry. FAME species were identified relative to a FAME mix C4‐C24 standard (Sigma‐Aldrich).

### Cellular NADPH Analysis

According to the manufacturer protocol, total cellular NADPH in 5‐day cultured cells with 0, 1, and 2 µM was quantified with Abcam's Colorimetric Total NADP and NADPH Assay Kit (ab186033). Samples were treated with provided lysis buffer (3:1) and centrifuged at 5000× *g* for 20 min at 20 °C. After subsequent incubation with the reaction mixture for 1 h at dark, absorbance was read at 460 nm with a GENESYS 150 UV‐vis spectrophotometer (Thermo Fisher Scientific Inc., Australia). The concentration of NADPH was quantified from the calculated NADPH standard curve.

### Total Protein Measurement

Total protein in 0, 1, and 2 µM CN‐TPAQ‐PF_6_ treated 7‐day cultured cells was determined by using the Bradford method.^[^ ^54^ ^]^ Samples were prepared according to the previous report^[^ ^55^ ^]^ with slight modifications. 10 mg of lyophilized samples were grounded with 5 mL of protein extraction buffer. After sonication, cells were centrifuged at 4000 × *g* for 20 min at 4 °C and supernatants were collected for analysis. The optical density of the samples was measured on a spectrophotometer (GENESYS 150 UV‐vis spectrophotometer, Thermo Fisher Scientific Inc., Australia) at 595 nm against the blank. Total protein was calculated from a prepared standard curve using bovine serum albumin.

### Data Analysis

The confocal microscopy data were analyzed with ImageJ 1.52a software.^[^ ^56^ ^]^ In brief, the raw images were exported to ImageJ in TIFF format. After subtracting the background from each image using a sliding paraboloid, the fluorescence channel was calculated to determine the area, area fraction means of grey values, and integrated density. The relative fluorescence intensity per cell was determined from the total cells by subtracting the background IntDen value from the IntDen value. FAME data were analyzed using the Agilent MassHunter Qualitative Navigator software. Differences between the control and other treatments were calculated with SPSS statistics software (version 23) at the *p < 0.05 and **p < 0.01 level through the paired sample *t*‐test. All the experiments were conducted with replications (n = 3).

## Conflict of Interest

The authors declare no conflict of interest.

## Author Contributions

S.R. performed conceptualization, investigation, data curation, and methodology, and wrote the original draft; AHM M.R. performed conceptualization, investigation, and wrote the original draft and reviewed and edited the manuscript, also performed data curation; B.D. performed data curation, investigation, and wrote the original draft and reviewed and edited the final manuscript; J.W. performed investigation and data curation; Y.T. performed conceptualization, supervision, and wrote the original draft and reviewed and edited the final manuscript; J.Q. performed conceptualization, supervision, and wrote the original draft and reviewed and edited the manuscript. All authors contributed to the final manuscript.

## Supporting information

Supporting Information

## Data Availability

The data that support the findings of this study are available on request from the corresponding author. The data are not publicly available due to privacy or ethical restrictions.
