# Supplementary material for: Aggregation‐Induced Emission Photosensitizer Boosting Algal Growth and Lipid Accumulation
Source: Small. 2024 Aug 19;20(47):2402463. doi: 10.1002/smll.202402463 (PMC11579964; doi:10.1002/smll.202402463)
Supplement: Supplementary file 1 — Supporting Information [file SMLL-20-2402463-s001.docx]

**Aggregation-Induced Emission Photosensitiser Boosting Algal Growth and Lipid Accumulation**

*Sharmin Rakhi, AHM-Mohsinul Reza, Brynley Davies, Jianzhong Wang, Youhong Tang ^*^, Jianguang Qin* ^*^

*Corresponding authors: [youhong.tang@flinders.edu.au](mailto:youhong.tang@flinders.edu.au) (Y Tang); [jian.qin@flinders.edu.au](mailto:jian.qin@flinders.edu.au) (J Qin)

**Determination of the autofluorescence of *C. reinhardtii***

In *C. reinhardtii,* chlorophylls and other biomolecules exhibit strong autofluorescent properties and may cause interference with lipid droplets (LDs) during fluorescent staining. Before selecting an appropriate AIE-based nanoprobe for lipid imaging, the autofluorescent spectrum of *C. reinhardtii* was determined with a fluorescent spectrophotometer (Cary Eclipse, MY17180002, Agilent Technologies, CA, USA) to minimise the background noise. Cells were excited at 350, 405 and 488 nm. The emission peak at around 400 nm for the excitation of 350 nm was unclear (Figure **S1a**). However, two emission peaks at 470 and 685 nm for the excitation of 405 nm (Figure **S1b**) were supposed to be due to the number of redox ratios (NAD(P)H/FAD) and autofluorescence of chlorophyll.^[1,2]^ However, maximum autofluorescence of chlorophyll was observed as a single peak when excited at 488 nm (Figure **S1c**).

| 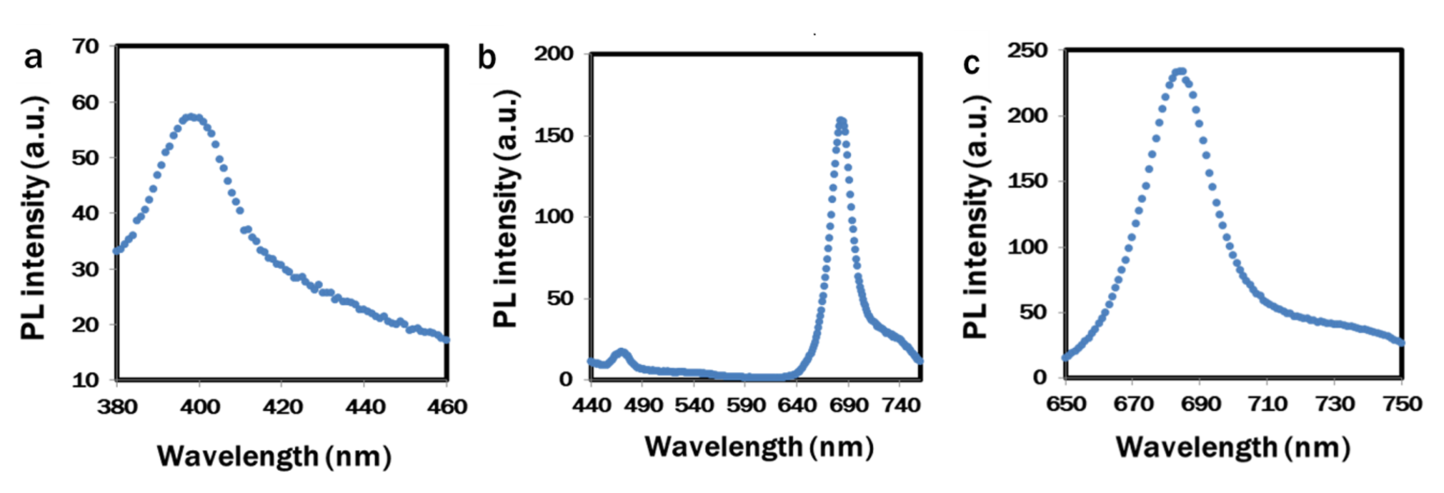 |
| --- |
| Figure **S1**. Autofluorescence of *C. reinhardtii* excited at different wavelengths (a) *λex:* 350 nm; (b) *λex:* 405 nm; (c) *λex*: 488 nm.  **Synthesis of CN-TPAQ-PF_6_**  To a stirred solution of compound CN-TPAQ (0.4 g, 1 mmol) in acetonitrile (10 mL) under nitrogen atmosphere, iodomethane (0.5 mL) was added and then heated to reflux for 16 h. The mixture was poured into diethyl ether to get the precipitates, then the precipitates were filtered and redissolved in 20 mL methanol, followed by adding saturated KPF6 solution (2 mL). After stirring for 2 h, evaporated the solvent and filtered the residue again, the residue was purified via flash column chromatography on silica gel (DCM: CH_3_OH, 10:1, v/v) to get the product as red solid with the yield of 85%. ^1^HNMR (400 MHz, DMSO-*d_6_*) δ 9.51 (d, *J* = 6.1 Hz, 1H), 8.52 (d, *J* = 8.9 Hz, 1H), 8.40-8.38 (m, 1H), 8.35-8.30 (m, 2H), 8.17 (d, *J* = 6.1 Hz, 1H), 8.08 (t, *J* = 7.7 Hz, 1H), 7.92 (d, *J* = 9.0 Hz, 2H), 7.75 (d, *J* = 8.5 Hz, 2H), 7.54 (t, *J* = 7.8 Hz, 2H), 7.47 (d, *J* = 8.5 Hz, 2H), 7.37 (dd, *J* = 13.7, 7.4 Hz, 3H), 7.12 (d, *J* = 8.9 Hz, 2H), 4.66 (s, 3H); ^13^C NMR (101 MHz, DMSO-d_6_) δ 159.92, 157.32, 152.65, 149.51, 147.73, 145.04, 139.36, 135.51, 133.48, 132.28, 131.49, 130.82, 130.61, 128.77, 127.77, 127.41, 127.22, 125.98, 124.52, 122.34, 120.05, 115.64, 114.73, 45.70。HRMS (MALDI): m/z ([C_32_H_23_N_4_]^+^): 463.1917; found: 463.1911.  **Synthesis of TPE-BO**  TPE-Br (0.982 g, 2mmol), bis(pinacolato)diboron (1.27 g, 5 mmol), potassiumacetate (1.374 g, 14 mmol), Pd(dppf)Cl2(80 mg, 0.11 mmol),and 30 mL of anhydrous DMF was added to a 50 mL flask. The reaction was heated at 85°C for 24 h under nitrogen. The dark brown reaction was cooled to room temperature, solvent evaporated, and then extracted with DCM. The organic layer was washed with deionized water and dried over anhydrous magnesium sulfate. After filtration and solvent evaporation, the residue was purifiedby silica gel column chromatography, using petroleum etherand ethyl acetate (*v/v*= 15:1) as eluent.^1^H NMR (300 MHz,CDCl_3_)δ7.54 (d,*J*= 1.5 Hz, 2H), 7.51 (d,*J*= 1.5 Hz, 2H),7.06−7.10 (m, 6H), 7.05−6.99 (m, 8H), 1.32 (s, 24H).^13^CNMR (100 MHz, CDCl_3_)δ146.80, 146.61, 143.61,143.45,141.34, 134.23, 134.14, 131.41, 130.76, 127.79, 127.66, 126.62,126.55, 83.78, 24.99.11B NMR (400 MHz, CDCl_3_)δ6.10.HRMS (m/z): ([M + H]^+^) calcd for C_38_H_42_B_2_O_4_, 585.3354; found, 585.3328  **Synthesis of 2-DPAN**  Benzophenone from cheap commercial product was refluxed with excess hydrazine hydrate in ethanol for 4 h to afford white needle crystals of M1 in 95% yield after cooling to room temperature. The Compounds M1 (7.85 g, 40 mmol) and 1-hydroxy-2-naphthaldehyde (7.23 g, 42 mmol) were dissolved with THF, ethanol and 2 drops of glacial acetic acid. The reaction was carried out for 4 hours under reflux. The residual solvent was removed by vacuum distillation and then purified by silica gel chromatography with DCM: PE (1:6) as eluent to afford 2-DPAN with nearly 90% yields. ^1^H NMR (CDCl_3_, 500 MHz), (TMS, ppm): 12.44 (s, 1H), 8.93 (s, 1H), 8.27-8.25 (d, J = 10.0 Hz, 1H), 7.79-7.77 (d, J = 10.0 Hz, 2H), 7.73-7.71 (d, J = 10.0 Hz, 1H), 7.56-7.50 (m, 4H), 7.47-7.36 (m, 6H), 7.33-7.29 (m, 2H).  **Cytotoxicity analysis of CN-TPAQ-PF_6_**  To determine the cytotoxicity of CN-TPAQ-PF_6_ in living cells, 3-(4,5-dimethyl2-thiazolyl)-2,5-diphenyltetrazolium bromide (MTT) assay was performed according to the previous report.[^3]^ with slight modification. HaCaT cell lines were cultured in 1 and 2 µM of CN-TPAQ-PF_6_ as treatment and 0.2% DMSO as a control for 24 hr. The optical density (OD) was measured by enzyme-linked immunosorbent assay (ELISA) at 490 nm. The cell inhibitory rate was determined by using the following formula:  Cell inhibitory rate (%) = (1 – OD treatment/OD control) × 100  In our experiment, CN-TPAQ-PF_6_ was biocompatible with *C. reinhardtii* at 1 µM and 2 µM as the growth rate increased at those concentrations. However, growth and lipid production declined when the cells were cultured with 3 µM CN-TPAQ-PF_6_ Therefore, 1 µM and 2 µM CN-TPAQ-PF_6_ concentrations were chosen to further determine the cytotoxicity on HaCaT cell lines with MTT assay. Compared to control, 100% cell viability was found in both 1 µM and 2 µM CN-TPAQ-PF_6_ treated cells, indicating high biocompatibility of this photosensitiser on living cells (Figure **S2**).   \| 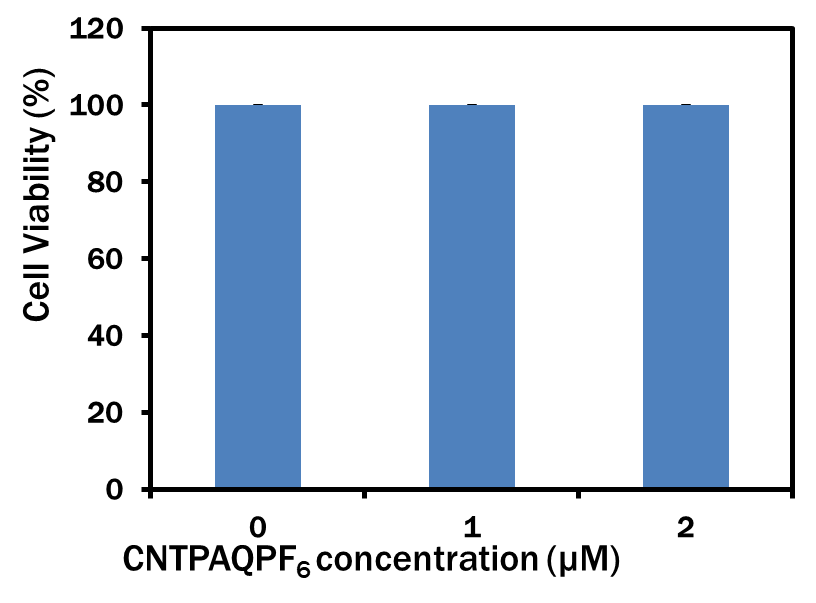 \| \| --- \| \| Figure **S2**. Cell viability (%) of HaCaT cell incubated with 1 µM and 2 µM CN-TPAQ-PF_6_ for 24 hr before MTT Assay. \| |

**GC-MS analysis of CN-TPAQ-PF_6_**

To detect the residues of CN-TPAQ-PF_6_, GCMS analysis was performed on day-7 with 2 µM CN-TPAQ-PF_6_ and compared with the initial day peak with the following conditions - Instrument: Waters Synapt HDMS, Capillary voltage: 2.25 kV, Ionization mode: ESI positive, Mass range: 50-1000m/z, Source Temp: 100 ^0^C, Desolvation temp: 300 ^0^C, Desolvation gas flow rate: 500 L/hr, Sampling cone voltage: 20 V, Extraction cone voltage: 4 V. Samples were dissolved in MeOH. At day 0, peaks were observed at the expected mass (Expected [M+H]+ mass 463.1923, observed mass 463.1790) (Figure **S3**), whereas no peaks occurred on day 7 (Figure **S4**). This might be due to the complete degradation of this molecule, which means no residues remain in the samples.

| 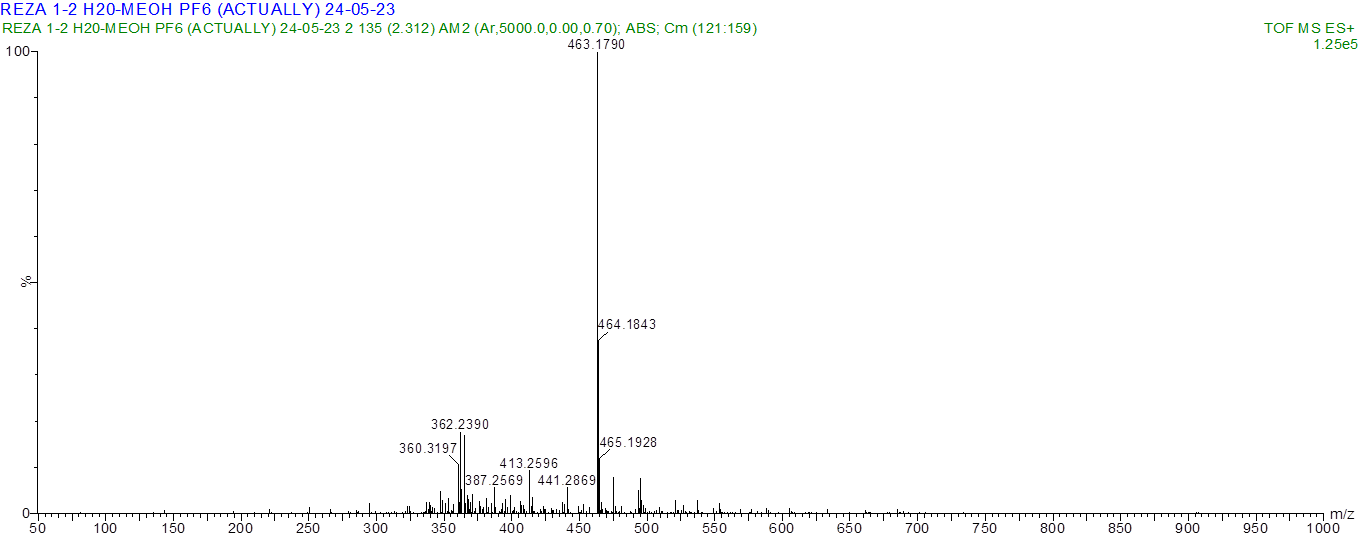 |
| --- |
| Figure **S3**. GCMS analysis of 2 µM TPA-A at day 0. Samples were prepared in MeOH. |

| 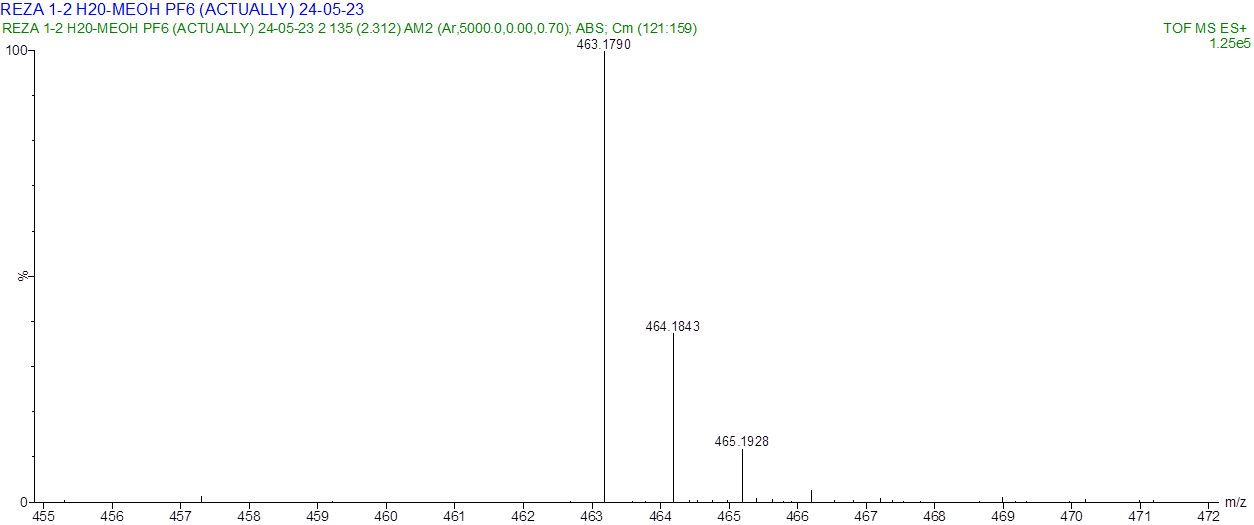 |
| --- |
| Figure **S4**. GCMS analysis of 2 µM TPA-A at day 7. Samples were prepared in MeOH. |

**References**

[1] Y. Wu, J.Y. Qu, *J. Biomed. Opt*. **2006**, *11*, 054023.

[2] A. H. M. M. Reza, S. F. Rakhi, X. Zhu, Y. Tang, J. Qin, *Biosensors*. **2022**, *12*, 208.

[3] A. Zerboni, R. Bengalli, G. Baeri, L. Fiandra, T. Catelani, P. Mantecca, *Nanomaterials*. **2019**, *9*, 1302.
